# Renal Functional Outcomes in Robot-Assisted Partial Nephrectomy with Minimum Layer Resection Using Virtual Three-Dimensional Image Assistance

**DOI:** 10.3390/jcm14207133

**Published:** 2025-10-10

**Authors:** Shuji Isotani, Tomoki Kimura, Taiki Ogasa, Takuro Kobayashi, Ippei Hiramatsu, Takeshi Ieda, Toshiyuki China, Fumitaka Shimizu, Masayoshi Nagata, Yuki Nakagawa, Hisamitsu Ide, Shigeo Horie

**Affiliations:** Department of Urology, Graduate School of Medicine, Juntendo University, Tokyo 113-8421, Japanm-nagata@juntendo.ac.jp (M.N.);

**Keywords:** robot-assisted partial nephrectomy (RAPN), three-dimensional virtual partial nephrectomy (3DvPN), Minimum Layer Resection (MLR) method

## Abstract

**Background**: Robot-assisted partial nephrectomy (RAPN) is a standard approach for localized renal cell carcinoma (RCC), emphasizing renal functional preservation. The Minimum Layer Resection (MLR) method, guided by 3D virtual partial nephrectomy (3DvPN) planning, was developed to balance oncological safety with parenchymal preservation. This study evaluated functional and oncological outcomes of RAPN with MLR and identified predictors of renal functional decline. **Methods**: We retrospectively analyzed 237 patients (after screening 312 cases) who underwent RAPN between 2012 and 2022 with ≥36-month follow-up. 3DvPN planning was used to guide MLR when feasible; both MLR and non-MLR were available and applied throughout the study period according to predefined indications. The primary endpoint was the percentage of estimated glomerular filtration rate (eGFR) preservation at 36 months; a ≥10% decline was clinically significant. Secondary endpoints included perioperative outcomes, acute kidney injury (AKI), and oncological outcomes such as margin involvement and recurrence. **Results**: The median age was 60 years, tumor size 29 mm, and warm ischemia time 21 min, with selective or superselective clamping achieved in 62.8% of cases. Postoperative AKI occurred in 25.0% (no patient required dialysis). At 3 years, the median eGFR preservation rate was 84.4%, and 28.5% of patients experienced a ≥10% decline. Independent predictors of short-term decline (14 days) were BMI > 25 kg/m^2^, AKI, and WIT > 25 min, whereas long-term decline (36 months) was associated with tumor size > 30 mm and WIT > 25 min. Margin involvement was 1.7%, recurrence 3.8%, and major complications (Clavien–Dindo ≥IV) occurred in 1.7%. **Conclusions**: In conclusion, RAPN with the MLR technique under 3DvPN guidance demonstrated favorable perioperative outcomes, acceptable oncologic safety, and good mid-term renal functional preservation (up to 36 months). The approach provides a reproducible surgical strategy that maximizes parenchymal preservation while maintaining negative surgical margins. Prospective multicenter studies with longer follow-up are warranted to confirm long-term durability and to define the role of MLR in routine practice.

## 1. Introduction

Partial nephrectomy (PN) is the gold standard treatment for localized renal cell carcinoma (RCC), particularly for T1 tumors, as it achieves equivalent oncological outcomes to radical nephrectomy while providing superior long-term renal functional preservation [1,2]. Postoperative renal function has been shown to depend primarily on the volume of preserved parenchyma, the duration of warm ischemia, and baseline renal function. Thompson et al. [3] reported that “every minute counts” when warm ischemia time (WIT) exceeds 25–30 min, while Mir et al. [4] confirmed that incremental ischemia directly correlates with nephron loss. Shikanov et al. [5] in a multicenter cohort, demonstrated that preserved parenchymal volume, more than ischemia time alone, is the strongest determinant of long-term renal outcomes. The introduction of robot-assisted partial nephrectomy (RAPN) has significantly enhanced surgical precision compared with conventional open or laparoscopic PN. RAPN allows refined dissection, facilitates selective arterial clamping, and enables meticulous renorrhaphy, translating into reduced blood loss, shorter ischemia times, and improved renal functional preservation [6,7,8,9]. These advantages have been confirmed in large series and systematic reviews, establishing RAPN as a preferred approach in many centers [7,8,9]. Recent advances in image-guided surgery have supported the use of three-dimensional (3D) reconstruction techniques in nephron-sparing surgery. Several studies have demonstrated that 3D reconstruction not only improves preoperative surgical planning and intraoperative anatomical orientation but also enhances surgeon confidence in delineating complex anatomical relationships, such as the proximity of tumors to the collecting system or segmental vessels [10,11,12]. By providing a patient-specific, high-fidelity representation of the renal anatomy, 3D models allow surgeons to anticipate technical challenges, choose the most appropriate resection plane, and optimize selective clamping strategies. Moreover, the use of 3D imaging during RAPN provides intraoperative feedback regarding tumor morphology, including subtle surface irregularities, tumor size, depth of parenchymal invasion, and spatial orientation relative to critical vascular or collecting structures. This real-time anatomical guidance enables surgeons to tailor the excision precisely along the actual contour of the tumor, thereby reducing unnecessary parenchymal sacrifice while maintaining an adequate oncological margin [13,14,15]. Such refined tumor-specific dissection supports the principle of nephron-sparing surgery by facilitating resection with minimal margins, which in turn contributes to maximal preservation of postoperative renal function (Isotani, Yazaki). Meta-analyses and contemporary reviews have further confirmed that RAPN, particularly when supported by 3D guidance, offers significant clinical advantages. These include shorter warm ischemia times, reduced intraoperative blood loss, and higher rates of trifecta or pentafecta achievement, as well as superior long-term functional preservation without compromising oncological safety [16,17,18]. Collectively, these findings emphasize that 3D-assisted RAPN represents not only a technological adjunct but also a shift toward more personalized and function-preserving kidney surgery. One of the key technical questions in RAPN concerns the method of tumor resection. Conventional resection typically includes removal of a visible rim of normal parenchyma surrounding the tumor, which provides oncological safety but sacrifices more healthy tissue. In contrast, simple enucleation (SE) follows the natural plane of the tumor pseudocapsule, aiming to maximize parenchymal preservation. This long-standing dichotomy highlights an important clinical issue, specifically the need to determine which approach offers the optimal balance between oncological safety and functional preservation. Dong et al. [19] reported that approximately 80% of tumors <7 cm are surrounded by a continuous pseudocapsule, suggesting that SE can often be performed safely. Minervini et al. [20] further showed that even when pseudocapsular invasion was present in 33.2% of cases, all patients still achieved negative surgical margins because of the presence of a thin (about 1 mm) layer of surrounding tissue. These findings support the oncological safety of SE; however, concerns remain regarding the potential risk of positive surgical margins and local recurrence. Conversely, conventional resection, while oncologically safe, is associated with increased blood loss, longer ischemia, and greater postoperative renal functional decline [11]. To balance these considerations, our group developed the Minimum Layer Resection (MLR) method, guided by three-dimensional virtual partial nephrectomy (3DvPN) planning, which targets a minimal (1–3 mm) margin beyond the pseudocapsule. This strategy aims to combine the oncological reliability of resection with the nephron-sparing advantage of SE.

In the present study, we evaluated the clinical and functional outcomes of RAPN performed with the MLR technique under 3DvPN guidance and sought to identify perioperative factors—including tumor characteristics, ischemia parameters, and surgical variables—that influence short- and long-term renal function.

## 2. Materials and Methods

**Figure 1:**
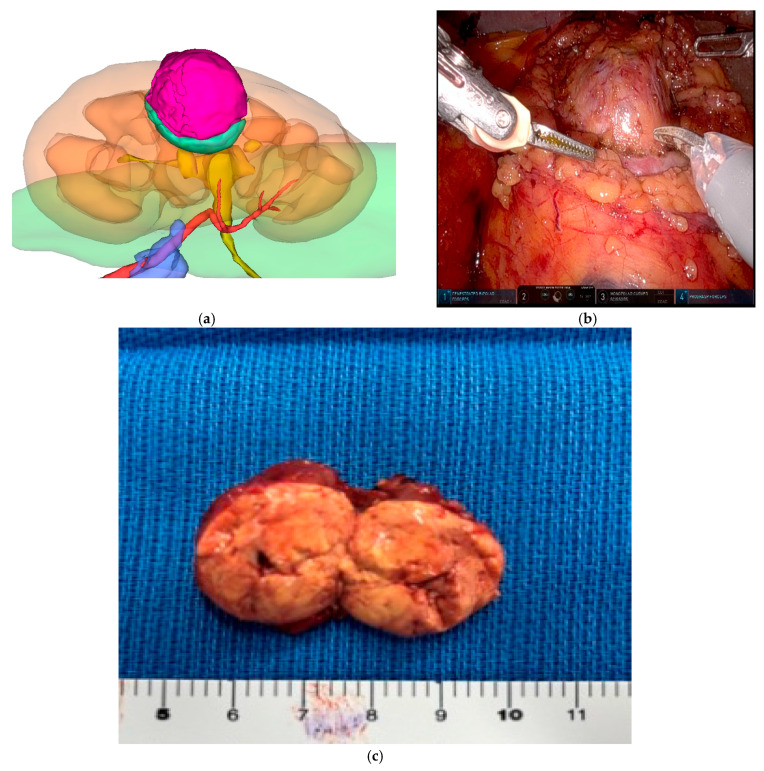
(**a**–**c**) Minimum layer resection method with virtual three-dimensional partial nephrectomy (3DvPN). (**a**). For the Minimum Layer Resection method, a preoperative Virtual 3D partial nephrectomy was served as a reference of Surgical Roadmap. (**b**). Tumor resection was performed with a 1–3 mm margin, precisely following its planned resection margin, and meticulously reproduced under direct visualization to ensure cancer control and optimal functional preservation. (**c**). Resected specimen by the Minimum Layer Resection method.

This was a retrospective, single-center study conducted at Juntendo University Hospital. A total of 312 consecutive patients with localized renal cell carcinoma (RCC) who underwent robot-assisted partial nephrectomy (RAPN) between January 2012 and December 2022 were screened; after excluding patients with re-do surgery, ureteropelvic junction obstruction, renal vein thrombus, conversion to radical nephrectomy, synchronous metastasis, concomitant surgery, or incomplete renal functional data, 237 patients constituted the final analytic cohort. The inclusion criteria were clinical stage T1a–T1b RCC with tumor size <7 cm and a minimum follow-up period of 36 months, while patients with a solitary kidney or prior ipsilateral renal surgery were excluded. All cases were reviewed at a multidisciplinary tumor board before surgery. The study protocol was approved by the Institutional Review Board of Juntendo University (Approval No. E23-0353, Date of Approval: 8 December 2023), and patient consent was obtained using an opt-out approach in accordance with institutional guidelines. All patients underwent contrast-enhanced multiphase computed tomography (CT), including unenhanced, corticomedullary, nephrographic, and excretory phases. Images were acquired at a slice thickness of 1.0 mm to enable high-resolution three-dimensional reconstruction. Three-dimensional virtual partial nephrectomy (3DvPN) models were generated using SYNAPSE VINCENT® software (versions 3–5; Fujifilm Corp., Tokyo, Japan), which automatically segmented the renal parenchyma, collecting system, tumor, and vasculature. Manual refinements were performed by the attending surgeon to ensure anatomical accuracy. Based on these reconstructions, the Minimum Layer Resection (MLR) protocol was pre-operatively planned, with the resection plane simulated along the interface between the tumor pseudocapsule and surrounding renal parenchyma, and a uniform 1–3 mm surgical margin was established. Patients were classified as MLR when they had exophytic tumors, preoperative contrast-enhanced CT suitable for 3D planning was available, and circumferential dissection along the minimum anatomic layer was considered feasible by the operating surgeon with 3DvPN guidance (MLR group, *n* = 163). All remaining RAPN cases were assigned to the non-MLR group, including completely endophytic lesions, procedures performed as planned simple enucleation without 3DvPN-guided MLR, and cases in which contrast-enhanced CT for 3D reconstruction was unavailable. All procedures were performed using a four-arm da Vinci Xi Surgical System (Intuitive Surgical, Sunnyvale, CA, USA) via a trans- or retro- peritoneal approach. Intraoperative image guidance was provided using the Tile-Pro display at the surgeon console, allowing real-time manipulation of the preplanned 3D vascular roadmap, which is depicted in Figure 1a–c. Selective or super-selective clamping of segmental or tertiary branches was performed whenever feasible, based on the preoperative 3D roadmap. In cases with multiple renal arteries, skeletonization was performed to identify the appropriate branch for clamping. In all cases, intraoperative ultrasound (Fujifilm Corp., Tokyo, Japan) was routinely performed prior to tumor excision using a laparoscopic/robotic probe to confirm tumor localization in accordance with the preoperative 3DvPN model, to verify the feasibility of circumferential dissection, to mark the minimal resection margin circumferentially, and to check the resection angle from all directions, thereby ensuring accurate execution along the planned layer. During tumor excision, the surgeon adhered strictly to the preoperative MLR plan; the MLR technique was defined as excision of the tumor along the natural pseudocapsule–parenchyma interface while maintaining the preplanned 1–3 mm surgical margin. After tumor excision, hemostasis of the resection bed was performed as an inner layer using running 3-0 V-Loc™ barbed hemostatic sutures (Medtronic, Minneapolis, MN, USA), followed by early unclamping. Additional focal hemostatic stitches were placed as required under a perfused field, and the parenchyma/capsule was then closed as an outer layer using 2-0 V-Loc™. At the end of the procedure, a fibrin sealant (Beriplast^®^; CSL Behring, Marburg, Germany) was sprayed over the resection surface before closure. Bleeding vessels were individually ligated as required, and sliding-clip stitches with 3-0 Vicryl sutures on SH-1 needles were used at the surgeon’s discretion, as described in our earlier reports [14,15,16]. Warm ischemia time (WIT) was recorded for all clamped cases; no prespecified WIT threshold was imposed for operative decision-making. Renal function was assessed using the estimated glomerular filtration rate (eGFR), which was calculated for each patient using the Chronic Kidney Disease Epidemiology Collaboration (CKD-EPI) equation, adjusted for age and sex. Serum creatinine levels were obtained at standardized time points: preoperatively (baseline), postoperative day 14, and at 1, 3, 6, 12, 24, and 36 months. The primary endpoint of renal function was defined as the percentage of eGFR preservation, calculated as postoperative eGFR divided by baseline eGFR × 100. A decline of ≥10% in eGFR from baseline was considered clinically significant functional deterioration. Acute kidney injury (AKI) was diagnosed according to the Kidney Disease: Improving Global Outcomes (KDIGO) criteria: an increase in serum creatinine of ≥0.3 mg/dL within 48 h, or an increase to ≥1.5 times the baseline value within seven days. Staging of AKI followed KDIGO definitions (Stage 1–3). Perioperative complications within 90 days were recorded and graded according to the Clavien–Dindo classification. Continuous variables were reported as medians with interquartile ranges (IQRs) and compared using the Mann–Whitney U test. Categorical variables were analyzed using chi-square or Fisher’s exact tests. Logistic regression analysis was used to identify independent predictors of short-term (14 days) and long-term (36 months) renal functional decline, defined as a ≥10% decrease in eGFR from baseline. Statistical significance was set at *p* < 0.05. All analyses were performed using JMP version 17.0 (SAS Institute Inc., Cary, NC, USA).

## 3. Results

**Figure 2:**
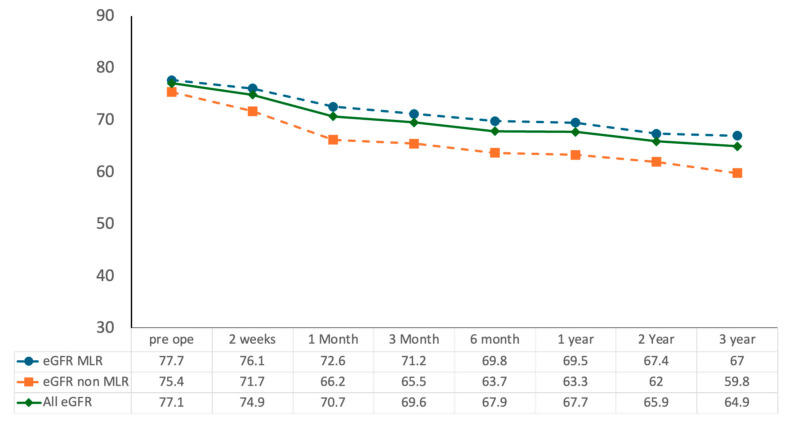
Longitudinal changes in renal function following RAPN with and without the MLR technique.

At a median follow-up of 42 months (IQR, 38–48), 237 patients were analyzed (MLR, *n* = 163; non-MLR, *n* = 74). Patient baseline characteristics are summarized in Table 1. The median age was 60.0 years (IQR, 51.0–69.0), and 77.6% were male. The median body mass index (BMI) was 24.2 kg/m^2^ (IQR, 22.2–26.7), with 36.5% categorized as overweight (BMI > 25 kg/m^2^). The median baseline estimated glomerular filtration rate (eGFR) was 76.6 mL/min/1.73 m^2^ (IQR, 64.6–90.1). Hypertension was present in 45.6%, diabetes mellitus in 14.3%, and cardiovascular disease in 9.6%. There were no significant differences between two groups in age (*p* = 0.52), sex (*p* = 0.39), BMI (*p* = 0.43), prevalence of hypertension (*p* = 0.93) or diabetes mellitus (*p* = 0.58), or baseline eGFR (*p* = 0.19). The median tumor size was 29 mm (IQR, 21–38), and the median RENAL nephrometry score was 7 (IQR, 6–9), reflecting an intermediate level of anatomical complexity. Patient baseline characteristics are summarized in Table 1. As expected from case selection, tumor complexity and stage differed between groups (RENAL distribution: MLR low 47.3%, intermediate 49.1%, high 3.7% vs. non-MLR 20.3%, 44.6%, 32.4%; *p* < 0.01), and T1a disease was more frequent in MLR (92.6% vs. 47.3%; *p* < 0.01). Perioperative outcomes are summarized in Table 2. Selective or super-selective clamping was performed in 62.8% of cases, while main artery clamping was required in the remainder. The median estimated blood loss was 50 mL (IQR, 20–100) in both groups (*p* = 0.89). The overall complication rate was 12.2% (*n* = 38), with Clavien–Dindo grade ≥IV major complications in 4 patients (1.7%). Notably, there were no between-group differences in surgical approach (intraperitoneal 66.9% vs. 67.6%; *p* = 0.42), estimated blood loss (both 50 mL; *p* = 0.89), or major complications (Clavien–Dindo ≥IV 1.2% vs. 2.7%; *p* = 0.65). Postoperative acute kidney injury (AKI), defined by KDIGO criteria, occurred in 59 patients (25%) overall and was less frequent after MLR (32/163, 19.8%) than after non-MLR (27/74, 36.5%; *p* < 0.01), no patient required dialysis for postoperative renal functional decline. This difference coincided with higher anatomical complexity and a tendency toward longer warm ischemia in the non-MLR cohort (Table 1 and Table 2). Histopathologic subtypes and ISUP grades are also summarized in Table 2; clear cell RCC was predominant, with other subtypes represented to a lesser extent. Renal functional outcomes demonstrated that the median eGFR preservation rate was 90.3% (IQR, 85.1–94.8) at 14 days and 84.4% (IQR, 77.5–89.6) at 36 months. A decline of ≥10% in eGFR was observed in 28.5% of patients (*n* = 89) at 36 months. The longitudinal course of renal function, expressed as the preservation rate of estimated glomerular filtration rate (eGFR), is depicted in Figure 2. A gradual, continued decline in eGFR beyond 36 months was observed, underscoring the need for longer-term follow-up. On multivariable logistic regression, BMI > 25 kg/m^2^ (odds ratio [OR] 1.87, 95% confidence interval [CI] 1.12–3.10, *p* = 0.016), postoperative AKI (OR 2.41, 95% CI 1.22–4.78, *p* = 0.012), and WIT > 25 min (OR 2.08, 95% CI 1.15–3.74, *p* = 0.015) were independent predictors of short-term renal functional decline, whereas tumor size >30 mm (OR 1.92, 95% CI 1.09–3.36, *p* = 0.023) and WIT > 25 min (OR 2.36, 95% CI 1.30–4.26, *p* = 0.004) were independent predictors of long-term decline. These analyses are summarized in Table 3a,b. Oncological outcomes were favorable. Surgical margin involvement occurred in 4 patients (1.7%), including 1 in the MLR group (0.63%) and 3 in the non-MLR group (3.95%). The median margin width from pathological results was 3.0 mm in the MLR group and 6.4 mm in the non-MLR group (*p* < 0.01). Representative cases of 3DvPN planning, intraoperative navigation, and pathological correlation are presented in Figure 1a–c. Local or distant recurrence was observed in 9 patients (3.8%), with 6 cases in the MLR group and 2 in the non-MLR group. No cancer-specific deaths were reported during follow-up. Given the limited number of oncologic events, between-group comparisons should be interpreted cautiously and considered hypothesis-generating.

Estimated glomerular filtration rate (eGFR, mL/min/1.73 m^2^) was assessed preoperatively and at 2 weeks, 1 month, 3 months, 6 months, 1 year, 2 years, and 3 years postoperatively. The MLR group showed significantly higher eGFR preservation compared with the non-MLR group throughout follow-up (* *p* < 0.01). Data are presented as median values.

This table summarizes the results of univariate and multivariate logistic regression analyses for predictors of renal functional decline at both 1 month (short-term) and 3 years (long-term) after RAPN with MLR. At 1 month, higher body mass index (BMI > 25 kg/m^2^), postoperative acute kidney injury (AKI), and warm ischemia time (WIT > 25 min) emerged as independent predictors of ≥10% decline in estimated glomerular filtration rate (eGFR). At 3 years, tumor size > 30 mm and WIT > 25 min were identified as significant long-term predictors.

## 4. Discussion

In this study, we evaluated the long-term renal functional and oncological outcomes of robot-assisted partial nephrectomy (RAPN) performed with the Minimum Layer Resection (MLR) technique under the guidance of three-dimensional virtual partial nephrectomy (3DvPN) planning. Our results demonstrated that MLR provided excellent renal functional preservation, with a median eGFR preservation rate of 84.4% at 36 months, while maintaining oncological safety with a low rate of surgical margin involvement (1.7%) and recurrence (3.8%). The longitudinal eGFR trajectory showed a gradual decline beyond 36 months, suggesting that mid-term preservation may not translate into durable long-term stability; therefore, extended follow-up is needed. The reproducibility of the MLR technique was also supported by pathological analysis of the resection margins. In our series, the median margin width was 3.0 mm in the MLR group compared with 6.4 mm in the non-MLR group, yet all cases with evaluable margins achieved negative surgical margins. These findings suggest that the integration of preoperative 3DvPN planning with intraoperative execution enabled surgeons to achieve a uniform, thin resection plane, thereby maximizing parenchymal preservation while maintaining oncological safety. Given the limited number of oncologic events, these findings should be interpreted as exploratory and preliminary, and broader adoption should await prospective, multicenter validation. One of the discussions in nephron-sparing surgery concerns the choice between simple enucleation (SE) and conventional resection. SE has been criticized for the potential risk of positive surgical margins or local recurrence due to incomplete tumor removal. However, Dong et al. reported that approximately 80% of renal tumors smaller than 7 cm are surrounded by a continuous pseudocapsule, providing a natural dissection plane [16]. Minervini et al. further observed that although pseudocapsular invasion was present in 33.2% of cases, all patients achieved negative surgical margins because of the presence of a thin rim of surrounding parenchyma, typically about 1 mm [17]. These findings suggest that the pseudocapsule and peritumoral degenerative tissue can ensure the technical feasibility and oncological safety of SE. On the other hand, conventional resection, which includes removal of a wider rim of normal parenchyma, has been associated with increased blood loss, longer ischemia time, and greater postoperative renal functional decline. The oncological significance of margin status has also been emphasized in previous studies. Bernhard et al. analyzed predictors of ipsilateral recurrence after nephron-sparing surgery and demonstrated that patients with positive surgical margins had a significantly higher risk of local recurrence [18]. This finding highlights the importance of achieving negative margins even in techniques designed to maximize parenchymal preservation. Our MLR method seeks to integrate the strengths of both approaches, preserving renal function by minimizing parenchymal loss while ensuring oncological safety through a controlled, image-guided margin of 1–3 mm. In this study, routine intraoperative robotic ultrasound was applied circumferentially just before excision to confirm the planned minimal (1–3 mm) margin and the resection plane derived from 3DvPN, which likely contributed to a low positive-margin rate despite thin margins. The importance of ischemia in determining postoperative renal function has been well established. Thompson et al. [3] first demonstrated that warm ischemia time (WIT) exceeding 25–30 min significantly increased the risk of renal functional deterioration, introducing the concept that “every minute counts”, and Mir et al. confirmed this relationship, showing that incremental ischemia directly correlates with nephron loss [3,4]. Shikanov et al. in a multicenter cohort, emphasized that preserved parenchymal volume, more than ischemia time alone, was the strongest determinant of long-term renal outcomes [5]. Collectively, these landmark studies shaped the current understanding of renal functional outcomes following partial nephrectomy. In our analysis, WIT > 25 min was an independent predictor of both short- and long-term functional decline. While many prior studies suggested that WIT has limited impact on long-term renal function when adequate parenchymal preservation is achieved, our results imply that ischemia still plays a significant role, particularly in procedures such as MLR where the preservation of parenchyma is maximized. The higher AKI rate observed in the non-MLR cohort coincided with greater anatomic complexity and a tendency toward longer WIT, consistent with our multivariable finding that postoperative AKI and WIT > 25 min independently predict early functional decline. This may reflect the dual effect of moderate ischemia and preserved parenchyma, which highlights the importance of further developing strategies such as zero-ischemia or off-clamp RAPN to improve outcomes. Comparison with previous RAPN studies further supports our findings. Mir et al. showed that renal function after nephron-sparing surgery depends on both ischemia and preserved parenchymal volume [19]. Our study adds to this body of evidence, showing that the combination of MLR and 3DvPN guidance can provide incremental advantages in both renal function and oncological control. Because allocation to MLR versus non-MLR was not randomized and the two cohorts differed clearly in anatomic complexity—e.g., higher RENAL scores, larger size, and more endophytic tumors in the non-MLR group—between-group comparisons should be interpreted with caution. Both techniques were available and employed throughout the study window (January 2012–December 2022); therefore, observed differences are unlikely to reflect “era effects” and instead primarily mirror case selection based on tumor anatomy and the operating surgeon’s feasibility judgment under 3DvPN guidance. The increasing use of three-dimensional (3D) reconstruction of renal anatomy from CT images has significantly enhanced preoperative planning and intraoperative navigation in partial nephrectomy. Several studies have shown that patient-specific 3D models improve the surgeon’s understanding of tumor location, vascular anatomy, and the relationship with the collecting system, thereby facilitating surgical planning and selective clamping strategies [7,8,9]. In practical terms, 3D reconstructions enable the surgeon to confirm—during dissection—the otherwise unseen intrarenal topography and its spatial relationship to the renal pelvis, calyces, and segmental vessels [13]. Operating with this continuous, patient-specific spatial feedback allows the excision to track the true tumor contour as planned, supporting resection with the intended minimal margin while avoiding unnecessary parenchymal sacrifice or inadvertent entry into adjacent structures. Bianchi et al. reported that 3D digital reconstructions improved perioperative outcomes and reduced complications in partial nephrectomy, and a case–control study demonstrated similar benefits [7,20]. Bernhard et al. highlighted the role of personalized 3D-printed models as educational tools for both patients and trainees [21]. Recent advances have also extended to intraoperative applications. Makiyama et al. showed the feasibility of patient-specific navigation systems, and our experience underscores that integrating 3DvPN with routine intraoperative ultrasound provides real-time feedback on tumor surface relief, size, and depth, enabling contour-following excision with minimal margins while maintaining orientation to feeding vessels and the collecting system [22]. Furthermore, De Backer et al. validated a novel perfusion-zone algorithm, confirming the ability of 3D planning tools to optimize selective clamping strategies [23]. Collectively, these findings underscore the growing role of 3D reconstruction and complementary intraoperative imaging in enhancing the precision and safety of nephron-sparing surgery. Augmented-reality–specific approaches were not evaluated in our cohort and were not the focus of this work. Several limitations of this study should be acknowledged. First, this was a retrospective, single-center study, which may introduce inherent selection bias and limit the generalizability of the results. Second, while MLR and non-MLR were defined a priori, the observational design and nonrandom group allocation may have allowed residual confounding. Third, although mid-term functional preservation was favorable, the observed gradual decline in eGFR beyond 36 months indicates the need for longer-term follow-up to assess durability and late events. Fourth, the number of oncologic events (margin positivity and recurrence) was small, limiting statistical power for between-group comparisons. Fifth, renal function was evaluated exclusively using eGFR, which reflects global renal function but does not provide side-specific data. More detailed assessments such as nuclear renography or functional MRI would allow a more precise evaluation of differential renal recovery and compensation by the contralateral kidney. Sixth, our center’s ready interventional radiology (IR) access and routine postoperative imaging facilitated both detection and definitive management of vascular lesions via selective embolization; in settings without comparable IR resources, detection and timely control may be limited, potentially increasing the clinical impact of bleeding events [24]. Seventh, surgeon learning curve and experience were not separately analyzed and may have influenced outcomes. Eighth, the non-MLR cohort was relatively small (*n* = 74), which limits statistical power for between-group and subgroup comparisons and increases the risk of type II error.

Despite these limitations, our findings indicate that the MLR technique guided by 3DvPN can achieve excellent renal function preservation without compromising oncological safety. However, broader adoption should await prospective, multicenter validation to confirm reproducibility, define patient-selection boundaries, and determine long-term durability. This study underscores the clinical relevance of MLR as an intermediate approach between resection and enucleation in RAPN, while highlighting the need for further multicenter prospective validation.

## 5. Conclusions

In conclusion, robot-assisted partial nephrectomy using the Minimum Layer Resection (MLR) technique with three-dimensional virtual partial nephrectomy (3DvPN) guidance demonstrated favorable perioperative outcomes, acceptable oncological safety, and favorable renal functional preservation at 36 months. This approach provides a reproducible surgical strategy designed to maximize parenchymal preservation while minimizing the risk of positive surgical margins. Warm ischemia time remained a key determinant of both short- and long-term renal function, underscoring the continued importance of ischemia minimization in nephron-sparing surgery. Nevertheless, the retrospective, single-center nature of this study and the limited subgroup analyses restrict the generalizability of the findings. Future multicenter, prospective studies with longer follow-up are warranted to validate these results and to define the potential role of MLR as a standard surgical strategy in RAPN.

## Figures and Tables

**Table 1:** Patient demographics and tumor characteristics.

Number of Patients			Total (*n* = 237)	MLR (*n* = 163)	No MLR (*n* = 74)	*p* Value
Age, yrs, median (IQR)			60.0 (51.0–69.0)	59.0 (50.0–69.0)	60.0 (52.2–69.0)	0.52
Sex, (Male%)			184 (77.6%)	124 (76.1%)	60 (81.1%)	0.39
BMI, (kg/m^2^) median (IQR)			24.2 (22.2–26.7)	24.4 (22.4–26.9)	23.9 (21.8–25.7)	0.43
Hypertension (%)			45.6%	45.4%	45.9%	0.93
Diabetes mellitus (%)			14.3%	13.5%	16.2%	0.58
Pre ope renal function Estimated GFR, mL/min/1.73 m median (IQR)			76.6 (64.6–90.1)	76.6 (65.8–91.1)	76.7 (63.6–89.0)	0.19
Serum Cr median (IQR)			0.8 (0.7–0.9)	0.8 (0.7–0.9)	0.8 (0.7–0.9)	0.05
Tumor size (cm) median (IQR)			2.9 (2.1–3.8)	2.6 (2.0–3.3)	4.0 (2.7–4.5)	<0.01
Estimated tumor volume (cm^3^)			9.2 (4.9–18.6)	7.6 (4.0–12.8)	16.5 (8.5–24.2)	<0.01
Nephrometry score	Low risk (4–6)	Low risk (4–6)	92 (39.1%)	77 (47.3%)	15 (20.3%)	<0.01
	Intermediate risk (7–9)	Intermediate risk (7–9)	113 (48.0%)	80 (49.1%)	33 (44.6%)	
	High risk (10–12)	High risk (10–12)	30 (12.8%)	6 (3.7%)	24 (32.4%)	
Clinical T stage, *n*	T1a	T1a	184 (78.3%)	151 (92.6%)	35 (47.3%)	<0.01
	T1b	T1b	49 (20.9%)	12 (7.4%)	34 (45.9%)	
	T2	T2	2 (1.0%)	0	5 (6.8%)	
Follow up, months			66.5 (47.8–72.2)	58.4	67.5	0.35
Laterality, (right %)			113 (48.0%)	76	37	0.24
Solitary Kidney			4 (1.7%)	2 (1.2%)	2 (2.7%)	0.44

**Table 2:** Perioperative and oncological outcomes in patients undergoing RAPN with and without the MLR technique.

		Total (*n* = 237)	MLR (*n* = 163)	No MLR (*n* = 74)	*p* Value
Surgical approach	Intraperitoneal approach	159 (67.1%)	109 (66.9%)	50 (67.6%)	0.42
Console time (mins)		148.2 (120–176)	140 (113–163)	146 (125–192)	<0.01
Ischemic time (mins)		22 (15–22)	18 (14–24)	22 (21–29.5)	0.06
Estimated Blood Loss, (mL)		50 (20–100)	50 (20–100)	50 (24–100)	0.89
Major complication (Clavien–Dindo classification 4>)		4	2 (1.2%)	2 (2.7%)	0.65
Postoperative AKI		59 (25%)	32 (19.8%)	27 (36.5%)	<0.01
Histology	Clear cell carcinoma	257 (83.8%)	129 (79.1%)	61 (82.4%)	0.93
	Papillary	17 (5.6%)	13 (2.4%)	4 (5.4%)	
	Chromophobe	23 (7.6%)	16 (9.8%)	7 (9.4%)	
	Others	10 (3.3%)	5 (3%)	5 (6.7%)	
Resection margin (mm)		4	3.0	6.4	<0.01
Positive margin		4 (1.7%)	1 (0.63%)	3 (3.95%)	0.07
Recurrence		9 (3.8%)	6 (3.6%)	2 (2.8%)	0.61

**Table 3:** Univariate and multivariate logistic regression analysis of predictors for ≥10% renal function decline after RAPN with MLR.

(**a**)				
	**Univariate analysis**		**Multi variate analysis**	
	OR	*p* value	OR	*p* value
Age	0.43	0.29		
BMI	1.08	0.02	1.05	0.15
Preope eGFR	1.52	0.58		
Tumor size	1.91	0.42		
RENAL score	0.97	0.88		
EBL (Estimated Blood loss)	2.94	0.16		
Post ope AKI	7.15	<0.01	4.67	<0.01
WIT (Warm ischemia time)	363	<0.01	55.7	<0.01
MLR (Minimum layer resection)	0.58	0.08		
(**b**)				
	**Univariate analysis**		**Multi variate analysis**	
	OR	*p* value	OR	*p* value
Age	1.13	0.85		
BMI	0.97	0.55		
Preope eGFR	1.52	0.61		
Tumor size	10.4	<0.01	1.21	0.86
RENAL score		0.10		
EBL (Estimated Blood loss)	4.76	0.09		
Post ope AKI	1.69	0.13		
WIT (Warm ischemia time)	1.05	0.02	1.03	0.01
MLR (Minimum layer resection)	0.18	<0.01	0.178	<0.01

## Data Availability

The data that support the findings of this study are available from Juntendo University Hospital but restrictions apply to the availability of these data, which contain sensitive personal information and are therefore not publicly available. Data may be made available from the corresponding author upon reasonable request and with permission of the Institutional Review Board of Juntendo University.

## Abbreviations

The following abbreviations are used in this manuscript:

RAPN	Robot-assisted partial nephrectomy
PN	Partial nephrectomy
RCC	Renal cell carcinoma
MLR	Minimum Layer Resection
3DvPN	Three-dimensional virtual partial nephrectomy
WIT	Warm ischemia time
eGFR	Estimated glomerular filtration rate
AKI	Acute kidney injury
IRB	Institutional Review Board
IQR	Interquartile range
OR	Odds ratio
CI	Confidence interval
CKD	Chronic kidney disease
KDIGO	Kidney Disease: Improving Global Outcomes
